# Graded Structure in Sexual Definitions: Categorizations of Having “Had Sex” and Virginity Loss Among Homosexual and Heterosexual Men and Women

**DOI:** 10.1007/s10508-016-0905-1

**Published:** 2016-12-05

**Authors:** Ava D. Horowitz, Edward Bedford

**Affiliations:** School of Psychology, University of Lincoln, Brayford Pool, Lincoln, LN6 7TS UK

**Keywords:** Definitions of sex, Definitions of virginity loss, Sexual behavior, Graded structure, Sexual orientation

## Abstract

Definitions of sexual behavior display a robust hierarchy of agreement regarding whether or not acts should be classed as, for example, sex or virginity loss. The current research offers a theoretical explanation for this hierarchy, proposing that sexual definitions display graded categorical structure, arising from goodness of membership judgments. Moderation of this graded structure is also predicted, with the focus here on how sexual orientation identity affects sexual definitions. A total of 300 18- to 30-year-old participants completed an online survey, rating 18 behaviors for how far each constitutes having “had sex” and virginity loss. Participants fell into one of four groups: heterosexual male or female, gay male or lesbian. The predicted ratings hierarchy emerged, in which bidirectional genital acts were rated significantly higher than unidirectional or nonpenetrative contact, which was in turn rated significantly higher than acts involving no genital contact. Moderation of graded structure was also in line with predictions. Compared to the other groups, the lesbian group significantly upgraded ratings of genital contact that was either unidirectional or nonpenetrative. There was also evidence of upgrading by the gay male sample of anal intercourse ratings. These effects are theorized to reflect group-level variation in experience, contextual perspective, and identity-management. The implications of the findings in relation to previous research are discussed. It is suggested that a graded structure approach can greatly benefit future research into sexual definitions, by permitting variable definitions to be predicted and explained, rather than merely identified.

## Introduction

Research into sexual behavior definitions has tended to focus on rates of agreement about which acts “count.” The widely replicated approach presents multiple behaviors (e.g., vaginal intercourse, manual-genital contact) and asks whether or not each constitutes a particular sexual term, such as “having sex” (e.g., Sanders & Reinisch, 1999), virginity loss (e.g., Trotter & Alderson, 2007), or sexual partner (e.g., Randall & Byers, 2003). Across the behaviors presented, agreement rates fall into the following robust hierarchy, from most to least endorsed: vaginal intercourse; anal intercourse; oral-genital contact; manual-genital contact; contact with breasts/nipples; kissing (cf. Horowitz & Spicer, 2013). This hierarchy persists, even where percentages vary as a function of, for example, culture (e.g., Pitts & Rahman, 2001; Randall & Byers, 2003), age (e.g., Sanders et al., 2010), target (e.g., Bogart, Cecil, Wagstaff, Pinkerton, & Abramson, 2000), or context (e.g., Trotter & Alderson, 2007). Qualitative research tends to support the definitional hierarchy (e.g., Carpenter, 2001; Mehta, Sunner, Head, Crosby, & Shrier, 2011).

Surprisingly, research often presents the agreement rate hierarchy without noting its hierarchical pattern (e.g., Bersamin, Fisher, Walker, Hill, & Grube, 2007; Randall & Byers, 2003). Alternatively, a hierarchy is noted but remains untheorized (e.g., Bogart et al., 2000; Peterson & Muehlenard, 2007). Byers, Henderson, and Hobson (2009) hypothesized (and found) significant differences between bidirectional (e.g., vaginal intercourse), unidirectional (e.g., genital touching), and no (e.g., oral contact with breasts/nipples) genital contact in the endorsement of behaviors as sex and as abstinence. However, no theoretical justification was given for these distinctions or for the pattern of results. Other research has noted that intercourse is more often classed as sex than oral-genital contact (e.g., Cecil, Bogart, Wagstaff, Pinkerton, & Abramson, 2002; Hans, Gillen, & Akande, 2010) but, again, theoretical explanations are absent. As a general rule, researchers have tended to focus on the implications of definitional disagreement. Practitioners, policy makers, and researchers are strongly cautioned to make clear and explicit the sexual acts they are targeting, in order to avoid misclassification biases (cf. Sanders et al., 2010). The current research aims to introduce a theoretical account for the endorsement hierarchy by conceptualizing sexual definitions as a matter of graded categorical judgment.

### Gender and Sexual Orientation Effects

A number of studies have tested for the effect of gender on sexual definitions, with highly inconsistent results. For example, Gute, Eshbaugh and Wiersma (2008) demonstrated significantly broader definitions of some acts by male participants, whereas in contrast, Trotter and Alderson (2007) found broader definitions by female participants and for differing subsets of the targeted acts. Other studies report a mixed pattern (e.g., Bersamin et al., 2007; Pitts & Rahman, 2001) or none at all (e.g., Byers et al., 2009; Randall & Byers, 2003). Significant gender effects have remained largely unexplained (e.g., Sanders & Reinisch, 1999; Trotter & Alderson, 2007). Moreover, where explanations are forthcoming, they tend to be post hoc and theorized in isolation from wider inconsistencies across the field (e.g., Gute et al., 2008; Pitts & Rahman, 2001).

In contrast, sexual orientation effects on sexual definitions have, until recently, been largely overlooked. Richters and Song’s (1999) 5% sample of “nonheterosexual” participants endorsed more behaviors as having sex than their heterosexual participants, although not significantly more. Similarly, Carpenter (2001) reported broader definitions for virginity loss among “nonheterosexual” than heterosexual participants. Meanwhile, Hill, Rahman, Bright, and Sanders (2010) studied “homosexual/gay” British and US men and found anal intercourse to be the highest rated act for both groups, rated significantly above vaginal intercourse.

Horowitz and Spicer’s (2013) study was the first to go beyond a simple contrast between the definitions of “heterosexuals” and “nonheterosexuals.” Ratings of having sex by a group of lesbians were compared to those by heterosexual males and females. For every listed act involving unidirectional genital contact, the lesbian group gave significantly higher ratings than the other two groups. Meanwhile, no significant group differences emerged between the two heterosexual groups or for any of the intercourse or nongenital contact acts investigated.

### Theoretical Accounts for Variability in Sexual Definitions

Where theoretical explanations for patterns in sexual behavior judgments have been offered, they have so far focused only on the variability, as opposed to the hierarchy, of sexual definitions. Taking a sociocultural perspective, Faulkner (2003) concluded that multiple, potentially contrary sexual scripts (cf. Simon & Gagnon, 1986, 1987) will vary in line with cultural, interpersonal, and intrapsychic frames of reference. Carpenter (2001) combined the sociocultural with the strategic, theorizing social constructions of virginity loss at a cultural level, which are actively embraced or resisted by individuals in accordance with personal and group-based concerns. Along similar lines, Horowitz and Spicer (2013) argued that “sexual definitions involve moral, cultural, and identity management dimensions.” The strategic dimension of sexual definitions was emphasized by Peterson and Muehlenhard (2007). They argued that sexual definitions are inevitably motivated, consequence-sensitive, and interest-advancing. Meanwhile, Gute et al. (2008) explained definitional discontinuities in their research as resulting from self-serving motives associated with the fundamental attribution error (cf. Ross, 1977).

### Applying a Graded Structure Approach to Sexual Definitions

In the current research, we propose a theoretical account for both the variability and the hierarchy of sexual behavior definitions, drawing on cognitive psychology research into categorization processes. The prototype approach has enriched the understanding of a broad range of fields, including social (e.g., Berthold, Leicht, Methner, & Guam, 2013; Harasymchuk & Fehr, 2013), forensic (e.g., Hoff, Rypdal, Mykletun, & Cooke, 2012; Smith, 1991), and clinical research (e.g., Feinstein, Meuwly, Davila, Eaton, & Yoneda, 2015; Hofsess & Tracey, 2010). The prototype approach is, however, merely one manifestation of the more encompassing graded structure approach within the categorization literature. Research within the wider tradition consistently demonstrates that categorization involves “graded structure” (Rosch & Mervis, 1975). Graded categorical structure occurs when some exemplars of a category are judged as better members than others (e.g., robin vs. ostrich as examples of the category “bird”).

The graded structure approach predicts graded structure to sexual definitions, in line with previous findings for other types of category (e.g., Barsalou, 1985; Rosch, 1974, 1975). Horowitz and Spicer (2013) represent the first support of such a prediction, demonstrating a hierarchy of means in ratings of what constitutes having sex. This means hierarchy was notably similar to the robust endorsement hierarchy of previous research (e.g., Sanders & Reinisch, 1999; Sanders et al., 2010). Rosch (1974, 1975) classically demonstrated that judges tend to agree about which category members are better than which others but vary in where they place category boundaries. The graded structure approach thus offers a way to explain the endorsement hierarchy of sexual behaviors. According to this perspective, it results from goodness of membership judgments of sexual behaviors and demonstrates disagreements about where in the hierarchy inclusion should cease, such that progressively fewer participants choose a “yes” response the further down the hierarchy of sexual behaviors one progresses.

Although consistency of agreement about graded structure is at the core of the categorization literature (Rosch & Mervis, 1975), variability in categorization judgments (beyond category boundary placement) has been both hypothesized and demonstrated. Most notably, Barsalou (1987) emphasized the variation in goodness of membership judgments that result from context and from individual differences in experience, episodic knowledge, and motivation. Hampton (2007) and Verheyen and Storms (2013), likewise, distinguished potential drivers of variable categorization, including intercontextual variation in the placement of category boundaries and differential prioritization of membership criteria. Consequently, although the primary aim of the current research was to identify a generic pattern of graded structure in sexual definitions, a preliminary investigation of definitional variability was also undertaken. The focus of the present study in this respect was upon definitional variability in line with sexual orientation identity.

Although Horowitz and Spicer (2013) demonstrated broad agreement as to the ratings hierarchy of sexual definitions, significantly higher ratings were found for judgments of unidirectional genital contact by the lesbian participants than the heterosexual male and female samples. Applying Barsalou (1987), this group-level variation may be expected to result from differences in the experiences and practices of lesbians in comparison with the other groups. Within the sexual behavior literature, there is indeed evidence of such differences (e.g., Blair & Pukall, 2014; Kinsey, Pomeroy, Martin, & Gebhard, 1953; Lever, 1995). Furthermore, differences have also been reported between lesbians and gay men (e.g., Blair & Pukall, 2014; Lever, 1995; Savin-William, 1990), and between gay and heterosexual men (e.g., Blair & Pukall, 2014). An additional site of experiential differences would be those associated with sexual minority versus sexual majority status (Cohen, Byers, & Walsh, 2008; Rothblum, 2000).

Work on context effects in the categorization literature is also very relevant here (for a review, see Yeh & Barsalou, 2006). Note, for example, Barsalou and Sewell’s (1984) demonstration that judging the typicality of an exemplar from different cultural points of view (e.g., of birds from an American vs. Chinese perspective) produced very different ratings (e.g., of robin vs. peacock). Similarly, when Vallée-Tourangeau, Anthony, and Austin (1998) investigated strategies of exemplar generation, strategies employing episodic knowledge were reported three times as often as strategies of purely semantic relation. Such findings suggest that, to the extent that different sexual orientation groups experience differing sexual behaviors, their concepts and definitions of sexual behavior are likely to differ.

### The Present Study

The current research applied a graded structure approach to sexual definitions. In partial replication of Horowitz and Spicer (2013), participants were tasked with rating a series of behaviors for how far each counts as having “had sex.” To expand the prior work, participants were also asked to rate the behaviors for how far they constitute virginity loss and a sample of gay men was added to the sexual orientation identity groups recruited. Two hypotheses were tested.

#### Graded Structure Hypothesis

The primary hypothesis, referred to henceforth as the graded structure hypothesis, predicted that definitional ratings of having “had sex” and virginity loss would exhibit graded structure, evidenced by consistently higher ratings for some sexual behaviors than others. Specific predictions were also made regarding which sexual behaviors were expected to receive higher versus lower ratings. Previous research into sexual definitions revealed intercourse acts, involving bidirectional genital contact and penetration, to be the most highly rated (Horowitz & Spicer, 2013) and highly endorsed (e.g., Randall & Byers, 2003; Sanders & Reinisch, 1999) sexual behaviors. Meanwhile, Byers et al. (2009) found significant differences in the endorsement of bidirectional, unidirectional and no genital contact acts as constituting sex. This led us to predict a ratings hierarchy in which bidirectional genital contact (e.g., vaginal and anal intercourse) would be rated significantly higher than unidirectional genital contact (e.g., oral-genital and manual-genital contact), which would, in turn, be rated significantly higher than behaviors involving no genital contact (e.g., touching breasts, kissing).

A hierarchy was also expected among the intercourse acts themselves. Previous findings place vaginal intercourse at the top of the ratings (Horowitz & Spicer, 2013) and endorsement hierarchies (e.g., Sanders & Reinisch, 1999; Trotter & Alderson, 2007). Meanwhile, Peterson and Muehlenard’s (2007) research attests to the equivocal status of “brief/partial intercourse.” We therefore predicted that vaginal intercourse would be rated above anal intercourse, while brief/partial intercourse would be rated lower than vaginal and anal intercourse acts described in the absence of qualifiers.

#### Moderation Hypothesis

A secondary hypothesis, referred to as the moderation hypothesis, was also tested. This predicted that generic graded structure in definitional ratings would exhibit group-level moderation. In the current research, modifications were expected to arise from the differing experiences and practices associated with the genital organ combinations involved in heterosexual, lesbian, and gay male partnerships. Specifically, it was predicted that the status of acts as having “had sex” and as virginity loss would be significantly upgraded for the closest acts to the (generically top-rated) vaginal intercourse exemplar that are congruent with a participant’s sexual orientation identity. The moderation hypothesis thus involves two predictions: Compared to the other groups, the lesbian participants would significantly upgrade ratings of unidirectional and nonpenetrative genital contact, and compared to the other groups, the gay male participants would significantly upgrade ratings of anal intercourse. Moderation may, however, also proceed from the operation of sexual orientation and gender separately. Thus, although the current study predicts differences on the basis of the interaction of gender and sexual orientation, each of these main effects was also tested.

Research into sexual definitions and behavior offers an additional foundation for the expectation of group-level moderation of generic graded structure. In particular, if sexual definitions are inevitably motivated, rhetorical, and consequence-sensitive (cf. Peterson & Muehlenard, 2007), variation in degree of membership judgments would be expected to follow from shared methods by which identity category membership (e.g., as a virgin, flirt, lesbian, gay man) may be embraced or resisted (cf. Carpenter, 2001; Faulkner, 2003). Such processes are expected to augment the variation in sexual definitions corresponding to sexual orientation identity.

## Method

### Participants

Participants were recruited to the online study via a brief standardized message. This was posted on a number of Web sites, online discussion groups and forums, and sent via e-mail to contacts of the second author. Selection of virtual venues and individuals was aimed so as to recruit a higher proportion of nonheterosexual participants than are represented in the general population. Thus, LGBT virtual communities were targeted, along with individuals who were known to the second author to self-identify as nonheterosexual. These were in addition to a range of virtual communities and known individuals with no particular sexual orientation self-identification profile.

In each case, the standardized message directed participants to an anonymous online survey. In total, 510 individuals accessed the survey. However, 61 of these individuals exited the survey without responding to any items, while a further 77 completed some or all of the demographic but none of the sexual definition items of the survey. Such individuals could not be included in the research, leaving an initial count of 372 participants.

With the objective of targeting young adults, the recruitment message and survey instructions expressed a particular wish for participants of 18–25 years of age. Nevertheless, 61 participants aged over 25 years completed the survey. To capitalize on this while retaining a focus on young adults, the age range for analysis was reset at 18–30 years old. Twenty-three additional participants were thereby eligible for inclusion. The remaining 38 older participants, and 19 participants who did not specify their age, were excluded from the study. Also excluded from the analysis, due to small numbers for comparison, were 15 participants who self-identified as other than heterosexual or homosexual, including bisexual, pansexual, and asexual individuals.

Thus, the final sample consisted of 300 individuals aged 18–30 (*M* age = 20.6, *SD* = 2.75), of which 191 were female and 109 were male, while 208 of these were heterosexual and 92 were homosexual. Consequently, the four groups who took part in the study were: 146 heterosexual women, 62 heterosexual men, 45 lesbians, and 47 gay men. Among the 295 participants who specified their nationality, 92.5% identified as British. Students made up 69% of the sample.

### Measures

#### Demographics

Five demographic items were coded: gender, age, sexual orientation, nationality, and whether or not the participant was currently a student.

#### Definitions of Sex

Definitions of sex were measured via a revision of the Sexual Behaviors Questionnaire (SBQ: Horowitz & Spicer, 2013). The SBQ posed the question: “Would you say you ‘had sex’ with someone if the most intimate behavior you engaged in was…” This was followed by a list of 13 sexual acts (adapted, in turn, from Pitts & Rahman, 2001) in conjunction with a six-point response scale, anchored by the two extremes of *definitely NOT sex* (1) and *definitely sex* (6). The acts included examples of intercourse (vaginal and anal), unidirectional genital contact (oral, manual, and with a sex aid), and breast/nipple contact, along with kissing. For the current study, the SBQ was revised to include five additional items. Three of these were autostimulation behaviors (derived from Randall & Byers, 2003), targeting masturbation while in the presence of, computer contact with, or telephone contact with another person. The remaining items captured two acts recurrently elicited when Peterson and Muehlenhard (2007) asked participants about ambiguous sexual experiences: “brief/partial penile-vaginal intercourse” and “nonpenetrative genital-to-genital contact.” The order of acts for the SBQ-R was randomly generated.

#### Definitions of Virginity Loss

Definitions of virginity loss were measured via the Virginity Loss Questionnaire (VLQ), which was identical to the SBQ-R but asked the question “Would you say you lost your virginity if the most intimate behavior you engaged in for the very first time was…” The same 18 sexual acts and response scales as the SBQ-R were then displayed, but accompanied by the anchors *definitely NOT virginity loss* (1) and *definitely virginity loss* (6).

#### Open-Comment Cue

An open-response cue offered space for participants to add any additional information or thoughts relating to the survey.

### Procedure

The standardized message inviting participation in the research outlined the study’s interest in beliefs about what acts count as having “had sex” and virginity loss. The focus on 18- to 25-year-olds and the anonymity of participation were also explained. Prospective participants were reassured that they would not be asked about their own sexual behavior but only about the extent to which they judged acts presented in a list to count as sex/virginity loss. The message ended with a direct Web link to the online survey.

At the survey site, an introduction largely repeated the recruitment message. Ethical approval for the study was notified, and a tick box item requested confirmation of both consent and that participants were at least 18 years of age. A decline of consent would take readers directly to the debrief page. The survey itself consisted of the following series of elements: a page of demographic questions; the SBQ-R; the VLQ; an open-comment cue; and a debrief.

### Analytic Plan

The current research predicted significantly higher ratings for intercourse acts than bidirectional genital contact acts and that acts involving no genital contact would be rated significantly lower than the other two classes of act. These predictions were derived from the endorsement patterns reported by Byers et al. (2009) for definitions of sex. However, the present study investigated definitions of virginity loss, in addition to sex, and employed a ratings methodology. Moreover, other works in the field (e.g., Horowitz & Spicer, 2013; Peterson & Muehlenard, 2007; Sanders & Reinisch, 1999) led us to also predict significant ratings effects for some individual items, specifically, among the intercourse acts. Consequently, before subjecting the findings to factorial analysis in order to test the hypotheses, we determined to establish a statistical grounding for any grouping of acts via principal components analysis (PCA).

## Results

### Principal Components Analyses of Definitional Responses

The scales for definitions of both sex and virginity loss were subjected to PCA with varimax rotation, following Kaiser–Meyer–Oklin’s and Bartlett’s tests demonstrating their appropriateness for PCA, sex *KMO* = .88, *χ* ^2^(153) = 4529.8, *p* < .001; virginity loss *KMO* = .90, *χ* ^2^(153) = 4535.9, *p* < .001.

For definitions of sex, the eigenvalues-greater-than-unit criterion (Kaiser, 1960) suggested a four-factor solution, with the following explanation of the variance: Factor 1, 48.89%; Factor 2, 11.63%; Factor 3, 6.94%; and Factor 4, 5.98%. Of the four cross-loading variables (factor loading >.40), three were allocated to the factor on which they loaded the highest, and the fourth on the basis of interpretability. This left only a single variable (vaginal intercourse) in Factor 4 (see Table 1). According to Cattell’s (1966) scree plot point-of-inflexion criterion, this factor should be excluded from further analysis.

**Table 1:** Principal components analysis of ratings for definitions of having “had sex”

Sexual behavior	Factor 1: Unidirectional/nonpenetrative contact	Factor 2: Nongenital contact	Factor 3: Other intercourse	Factor 4: Vaginal intercourse
Initiative oral-genital contact	**.86**			
Receptive oral-genital contact	**.85**			
Receptive sex aid contact	**.84**			
Initiative sex aid contact	**.84**			
Initiative manual-genital contact	**.81**			
Receptive manual-genital contact	**.79**	.41		
Nonpenetrative genital-to-genital contact	**.70**			
Simultaneous masturbation in another’s presence	.59	**.48**		
Initiative manual contact with breasts/nipples		**.90**		
Receptive manual contact with breasts/nipples		**.89**		
Initiative oral contact with breasts/nipples		**.86**		
Receptive oral contact with breasts/nipples		**.83**		
Deep kissing		**.72**		
Simultaneous masturbation via phone contact	.42	**.57**		.41
Simultaneous masturbation via computer contact		**.55**		.53
Anal intercourse			**.80**	
Brief/partial intercourse			**.75**	
Vaginal intercourse				**-.75**
Eigenvalues	8.80	2.09	1.25	1.08
Percentage of variance	48.89	11.63	6.94	5.98
Cronbach’s alpha^a^	.94	.92	.38	–

Factor loadings <.40 are suppressed. In boldface are factor loadings incorporated into the factor

^a^Cronbach’s alphas for incorporated items

For definitions of virginity loss, a three-factor solution was suggested by both the Kaiser and Cattell criteria. Factor 1 explained 50.11% of the variance, Factor 2 explained 13.25%, and Factor 3 6.27% of the variance. Only 2 of the 18 variables cross-loaded and were allocated to the factor on which they loaded the highest (see Table 2).

**Table 2:** Principal components analysis of ratings for definitions of virginity loss

Sexual behavior	Factor 1: Nongenital contact	Factor 2: Unidirectional/nonpenetrative genital contact	Factor 3: Intercourse
Initiative manual contact with breasts/nipples	**.92**		
Receptive manual contact with breasts/nipples	**.90**		
Initiative oral contact with breasts/nipples	**.84**		
Simultaneous masturbation via computer contact	**.80**		
Receptive oral contact with breasts/nipples	**.80**		
Deep kissing	**.77**		
Simultaneous masturbation via phone contact	**.75**		
Simultaneous masturbation in another’s presence	**.68**	.50	
Initiative oral-genital contact		**.85**	
Receptive oral-genital contact		**.84**	
Initiative sex aid contact		**.81**	
Receptive sex aid contact		**.80**	
Receptive manual-genital contact		**.78**	
Initiative manual-genital contact	.42	**.77**	
Nonpenetrative genital-to-genital contact		**.76**	
Brief/partial intercourse			**.73**
Anal intercourse			**.61**
Vaginal intercourse			**.46**
Eigenvalues	9.02	2.38	1.13
Percentage of variance	50.11	13.25	6.27
Cronbach’s alpha^a^	.94	.93	.26

Factor loadings <.40 are suppressed. In boldface are factor loadings incorporated into the factor

^a^Cronbach’s alphas for incorporated items

On further analysis, and in line with the graded structure hypothesis, two strong groupings of sexual behaviors emerged, each with high internal consistency on both scales: Unidirectional/Nonpenetrative Genital Contact (sex Factor 1, *α* = .94, and virginity loss Factor 2, *α* = .93) and Nongenital Contact (sex Factor 2, *α* = .92, and virginity loss Factor 1, *α* = .94). It was, therefore, determined that these two classes of sexual behavior could meaningfully be aggregated for further analysis. The factor structure of the remaining intercourse behaviors, however, suggested more caution with respect to aggregation.

### Act Type, Gender, and Sexual Orientation Effects

The research hypotheses and PCA results combined to recommend that definitions of sex and virginity loss be subjected to a two-stage analysis. Firstly, in order to test the graded structure hypothesis, an analysis was undertaken of the composite scores for intercourse, unidirectional/nonpenetrative genital contact, and nongenital contact. Such an analysis was considered warranted, despite PCA indicators suggesting caution about composite scoring of the intercourse behaviors. It also permitted testing of the moderation hypothesis prediction that unidirectional/nonpenetrative genital contact would be upgraded by the lesbian group, in comparison with the other groups in the study. Secondly, an analysis was undertaken of the discrete ratings for each of the three intercourse acts. This permitted testing of the moderation hypothesis prediction that anal intercourse ratings would be upgraded by gay male participants, in comparison with the other groups in the study. It also attends to issues with aggregating the intercourse acts, as highlighted by the PCA.

The research hypotheses were tested via three-way mixed analysis of variance (ANOVA) of each scale separately, with gender and sexual orientation as between-subjects variables, and act type as a within-subject variable. Significant interactions were further subjected to follow-up simple effect and *t* testing, applying a Bonferroni correction to *p* < .0017. Means and SDs for each separate act are shown in “Appendix.”

#### Comparison of Composite Scores: Intercourse, Unidirectional/Nonpenetrative Genital Contact, and Nongenital Contact

##### Definitions of Sex

For the definitions of sex ratings, a 2 (gender) × 2 (sexual orientation) × 3 (act type) mixed ANOVA was conducted on the composite scores for intercourse, unidirectional/nonpenetrative genital contact, and nongenital contact. As predicted by the graded structure hypothesis, the composite analysis revealed a highly significant effect of act type (see Table 3). Also significant were the main effects of sexual orientation, *F*(1, 256) = 13.70, *p* < .001, *η*_p_^2^ = .05, and gender, *F*(1, 256) = 7.57, *p* = .006, *η*_p_^2^ = .03. The two-way interactions of Act Type × Sexual Orientation, *F*(1.69, 433.06) = 39.42, *p* < .001, *η*_p_^2^ = .13, and of Act Type x Gender, *F*(1.69, 433.06) = 14.95, *p* < .001, *η*_p_^2^ = .06, were both significant, while the interaction of Gender × Sexual Orientation was nonsignificant.

**Table 3:** Composite scores analysis for definitions of sex and virginity loss as a function of act type: means, *SD*s, and test statistics within each sexual orientation identity group and in total

Definition and group	Means and SDs by act type			Within-subject effects	Within-subject linear contrasts
	Intercourse *M* (*SD*)	Unidirectional/nonpenetrative genital contact *M* (*SD*)	Nongenital contact *M* (*SD*)		
*Sex*					
Lesbian	5.41^ab^ (.73)	4.73^ac^ (.92)	1.88^bc^ (.75)	*F*(2, 78) = 385.58, *p* < .001, *η*_p_^2^ = .91	*F*(1, 39) = 682.76, *p* < .001, *η*_p_^2^ = .95
Gay male	5.67^ab^ (.46)	3.63^ac^ (1.10)	1.67^bc^ (.59)	*F*(1.67, 72.10) = 422.92, *p* < .001, *η*_p_^2^ = .91	*F*(1, 43) = 1437.25, *p* < .001, *η*_p_^2^ = .97
Heterosexual female	5.68^ab^ (.45)	3.23^ac^ (1.41)	1.78^bc^ (1.02)	*F*(1.63, 204.89) = 722.33, *p* < .001, *η*_p_^2^ = .85	*F*(1, 126) = 1616.13, *p* < .001, *η*_p_^2^ = .93
Heterosexual male	5.54^ab^ (.64)	2.86^ac^ (1.54)	1.63^bc^ (.94)	*F*(1.55, 74.34) = 278.59, *p* < .001, *η*_p_^2^ = .85	*F*(1, 48) = 715.58, *p* < .001, *η*_p_^2^ = .94
Total	5.61^ab^ (.55)	3.46^ac^ (1.44)	1.75^bc^ (.90)	*F*(1.69, 433.06) = 1316.39, *p* < .001, *η*_p_^2^ = .84	*F*(1, 256) = 3188.44, *p* < .001, *η*_p_^2^ = .93
*Virginity loss*					
Lesbian	5.39^ab^ (.80)	4.17^ac^ (1.14)	1.44^bc^ (.65)	*F*(2, 74) = 248.72, *p* < .001, *η*_p_^2^ = .87	*F*(1, 37) = 635.35, *p* < .001, *η*_p_^2^ = .95
Gay male	5.31^ab^ (1.04)	2.36^ac^ (1.24)	1.22^bc^ (.38)	*F*(2, 70) = 227.28, *p* < .001, *η*_p_^2^ = .87	*F*(1, 35) = 552.69, *p* < .001, *η*_p_^2^ = .94
Heterosexual female	5.36^ab^ (.74)	2.04^ac^ (1.22)	1.34^bc^ (.73)	*F*(1.58, 203.42) = 1019.23, *p* < .001, *η*_p_^2^ = .89	*F*(1, 129) = 1865.97, *p* < .001, *η*_p_^2^ = .94
Heterosexual male	5.04^ab^ (.90)	1.80^ac^ (1.29)	1.29^bc^ (.76)	*F*(1.57, 81.85) = 278.59, *p* < .001, *η*_p_^2^ = .89	*F*(1, 52) = 758.09, *p* < .001, *η*_p_^2^ = .94
Total	5.29^ab^ (.84)	2.35^ac^ (1.44)	1.33^bc^ (.68)	*F*(1.70, 430.29) = 1376.62, *p* < .001, *η*_p_^2^ = .85	*F*(1, 253) = 2938.57, *p* < .001, *η*_p_^2^ = .92

For each row titled “Lesbian,” “Gay male,” “Heterosexual female,” and “Heterosexual male,” the test statistics are from the simple effects analyses examining ratings differences as a function of act type within each sexual orientation identity group. For each row titled “total,” the test statistics are the main effects of act type (i.e., across all groups) from the omnibus ANOVA

^abc^ Within each row, means with the same letter were significantly different to a Bonferroni-corrected *p* < .0017

These findings were qualified by a significant three-way interaction of Act Type × Sexual Orientation × Gender, *F*(1.69, 433.06) = 7.22, *p* = .002, *η*_p_^2^ = 03. Simple effects analysis of act type for each of the four groups demonstrated highly significant differences for each, with very high effect sizes (see Table 3). As the graded structure hypothesis predicted, in all cases, the means for intercourse were significantly higher than those for unidirectional/nonpenetrative genital contact, which were significantly higher than those for nongenital contact. Highly significant linear contrasts were found for all four groups, demonstrating that ratings systematically decreased from intercourse to unidirectional/nonpenetrative genital contact to nongenital contact (see Table 3).

Within each act type, simple effects group comparisons revealed four significant findings, all with respect to unidirectional/nonpenetrative genital contact. In support of the moderation hypothesis, the lesbian group rated unidirectional/nonpenetrative genital contact as significantly more constitutive of sex than each of the other groups: the heterosexual males, *F*(1, 99) = 59.84, *p* < .001, *η*_p_^2^ = .38; the gay males, *F*(1, 87) = 28.13, *p* < .001, *η*_p_^2^ = .24; and the heterosexual females, *F*(1, 183) = 41.28, *p* < .001, *η*_p_^2^ = .18. Unexpectedly, the gay males also rated unidirectional/nonpenetrative genital contact significantly higher than the heterosexual males, *F*(1, 102) = 10.58, *p* = .0016, *η*_p_^2^ = .09.

##### Definitions of Virginity Loss

For ratings of virginity loss, a 2 (gender) × 2 (sexual orientation) × 3 (act type) mixed ANOVA was conducted on the composite scores for intercourse, unidirectional/nonpenetrative genital contact, and nongenital contact. Supporting the graded structure hypothesis, there was a significant main effect for act type (see Table 3). Also significant were the main effects of sexual orientation, *F*(1, 253) = 25.67, *p* < .001, *η*_p_^2^ = .09, and of gender, *F*(1, 253) = 20.90, *p* < .001, *η*_p_^2^ = 08. There were significant two-way interactions of Act Type × Sexual Orientation, *F*(1.70, 430.29) = 44.91, *p* < .001, *η*_p_^2^ = .15, and of Act Type × Gender, *F*(1.70, 430.29) = 21.17, *p* < .001, *η*_p_^2^ = .08, but not of Gender × Sexual Orientation.

These effects were qualified by a significant three-way interaction of Act Type × Sexual Orientation x Gender, *F*(1.70, 430.29) = 18.57, *p* = .002, *η*_p_^2^ = 07. Simple effects analysis of the four groups for act type all demonstrated highly significant differences for each, with very high effect sizes (see Table 3). As predicted by the graded structure hypothesis, in each case, intercourse was rated significantly higher than unidirectional/nonpenetrative genital contact, which, in turn, was rated significantly higher than nongenital contact. Linear contrasts demonstrated highly significant effects for each group, revealing a systematic decrease in ratings from intercourse to unidirectional/nonpenetrative genital contact to nongenital contact (see Table 3).

Within each act type, simple effects group comparisons revealed three significant findings, all with respect to unidirectional/nonpenetrative genital contact. As the moderation hypothesis predicted, the lesbian group rated unidirectional/nonpenetrative genital contact as significantly more constitutive of virginity loss than the three remaining groups: the heterosexual males, *F*(1, 95) = 75.14, *p* < .001, *η*_p_^2^ = .44; the gay males, *F*(1, 80) = 39.34, *p* < .001, *η*_p_^2^ = .33; and the heterosexual females, *F*(1, 172) = 84.33, *p* < .001, *η*_p_^2^ = .33.

#### Intercourse Behaviors: Vaginal, Anal, and Brief/Partial Intercourse

##### Definitions of Sex

Definitions of sex were subjected to a 2 (gender) × 2 (sexual orientation) × 3 (act type) mixed ANOVA of the intercourse behaviors. As the graded structure hypothesis predicted, among the intercourse behaviors, there was a significant main effect of act type (see Table 4). The main effects of gender and sexual orientation were nonsignificant, while among the two-way interactions, only the interaction of Gender × Sexual Orientation was significant, *F*(2, 572) = 7.93, *p* = .005, *η*_p_^2^ = .03.

**Table 4:** Intercourse behaviors analysis for definitions of sex and virginity loss as a function of act type: means, *SD*s, and test statistics within each sexual orientation identity group and in total

Definition and group	Means and SDs by act type			Within-subject effects	Within-subject linear contrasts
	Vaginal Intercourse *M* (*SD*)	Anal Intercourse *M* (*SD*)	Brief/partial intercourse *M* (*SD*)		
*Sex*					
Lesbian	5.95^a^ (.31)	5.48 (1.25)	4.83^a^ (1.32)	*F*(2, 82) = 14.14, *p* < .001, *η*_p_^2^ = . 26	*F*(1, 41) = 29.78, *p* < .001, *η*_p_^2^ = .42
Gay male	5.87^a^ (.73)	6.00^b^ (.00)	5.17^ab^ (.98)	*F*(2, 92) = 31.98, *p* < .001, *η*_p_^2^ = . 41	*F*(1, 46) = 32.00, *p* < .001, *η*_p_^2^ = .41
Heterosexual female	5.998^a^ (.15)	5.70^b^ (.80)	5.30^ab^ (1.01)	*F*(2, 278) = 26.72, *p* < .001, *η*_p_^2^ = .16	*F*(1, 139) = 53.44, *p* < .001, *η*_p_^2^ = .28
Heterosexual male	5.93^a^ (.40)	5.62^b^ (1.02)	5.00^ab^ (1.30)	*F*(2, 120) = 16.84, *p* < .001, *η*_p_^2^ = .22	*F*(1, 60) = 29.12, *p* < .001, *η*_p_^2^ = .33
Total	5.95^ab^ (.38)	5.70^ac^ (.87)	5.15^bc^ (1.13)	*F*(2, 572) = 73.54, *p* < .001, *η*_p_^2^ = .21	*F*(1, 286) = 130.91, *p* < .001, *η*_p_^2^ = .31
*Virginity loss*					
Lesbian	6.00^ab^ (.00)	5.07^a^ (1.73)	4.90^b^ (1.46)	*F*(2, 82) = 10.64, *p* < .001, *η*_p_^2^ = .21	*F*(1, 41) = 23.58, *p* < .001, *η*_p_^2^ = .37
Gay male	5.56 (1.35)	5.82^a^ (.82)	4.64^a^ (1.77)	*F*(2, 74) = 10.60, *p* < .001, *η*_p_^2^ = .22	*F*(1, 37) = 8.38, *p* < .001, *η*_p_^2^ = .19
Heterosexual female	5.85^ab^ (.81)	4.92^a^ (1.57)	5.29^b^ (1.08)	*F*(2, 264) = 20.74, *p* < .001, *η*_p_^2^ = .14	*F*(1, 132) = 23.28, *p* < .001, *η*_p_^2^ = .15
Heterosexual male	5.89^ab^ (.68)	4.77^a^ (1.74)	4.55^b^ (1.77)	*F*(2, 110) = 13.82, *p* < .001, *η*_p_^2^ = .20	*F*(1, 55) = 27.01, *p* < .001, *η*_p_^2^ = .33
Total	5.84^ab^ (.83)	5.04^a^ (1.58)	4.98^b^ (1.44)	*F*(2, 530) = 36.20, *p* < .001, *η*_p_^2^ = .12	*F*(1, 265) = 81.40, *p* < .001, *η*_p_^2^ = .24

For each row titled “Lesbian,” “Gay male,” “Heterosexual female,” and “Heterosexual male,” the test statistics are from the simple effects analyses examining ratings differences as a function of act type within each sexual orientation identity group. For each row titled “total,” the test statistics are the main effects of act type (i.e., across all groups) from the omnibus ANOVA

^abc^ Within each row, means with the same letter were significantly different to a Bonferroni-corrected *p* < .0017

These findings were, however, qualified by a significant three-way interaction of Act Type x Sexual Orientation x Gender, *F*(2, 572) = 3.30, *p* = .042, *η*_p_^2^ = .01. Simple effects analysis demonstrated act type to be significant for each group (see Table 4). Contrary to prediction, follow-up *t* tests did not reveal vaginal intercourse to be rated significantly higher than anal intercourse for any group. However, for all four groups, vaginal intercourse was rated significantly higher than brief/partial intercourse. For all the groups except the lesbian group, brief/partial intercourse was also rated significantly lower than anal intercourse. Partially supporting the graded structure hypothesis, for three of the four groups—the lesbian, heterosexual female, and heterosexual male groups—significant linear contrasts demonstrated a systematic decrease in ratings from vaginal to anal to brief/partial intercourse. Meanwhile, supporting the moderation hypothesis, among the group of gay males, a significant linear contrast pertained in the order anal to vaginal to brief/partial intercourse (see Table 4).

In contradiction to the moderation hypothesis, within each act type, simple effects group comparisons revealed no significant findings once the Bonferroni correction was applied. This was so, despite the gay males consistently rating anal sex with the maximum possible value, while the remaining groups demonstrated less extreme ratings (see Table 4).

##### Definitions of Virginity Loss

Ratings of virginity loss were submitted to a 2 (gender) × 2 (sexual orientation) × 3 (act type) mixed ANOVA of the intercourse behaviors. In line with the graded structure hypothesis, the main effect of act type was significant (see Table 4). Neither of the remaining main effects were significant, but Act Type x Sexual Orientation, *F*(2, 530) = 6.21, *p* = .002, *η*_p_^2^ = .02, and Act Type × Gender, *F*(2, 530) = 5.79, *p* = .004, *η*_p_^2^ = .02, were both significant.

All of these results were qualified by a significant three-way interaction of Act Type × Sexual Orientation × Gender, *F*(2, 530) = 4.51, *p* = .012, *η*_p_^2^ = .02. Simple effects analysis demonstrated a significant effect of act type for all four groups (see Table 4). In partial support of the graded structure hypothesis, follow-up testing revealed that vaginal intercourse was rated significantly higher than anal intercourse among three of the four groups—the exception being the gay male group. Vaginal intercourse was also rated significantly higher than brief/partial intercourse among these same three groups. In line with the moderation hypothesis, among the gay male group, anal intercourse was rated the highest of the three intercourse behaviors, and this was significantly higher than brief/partial intercourse. It was not, however, rated significantly higher than vaginal intercourse (see Table 4). In partial support of the graded structure hypothesis, linear contrasts demonstrating a systematic decrease in ratings from vaginal to anal to brief/partial intercourse were significant for the lesbian, heterosexual female, and heterosexual male groups. Meanwhile, in support of the moderation hypothesis, among the gay male group, a significant linear contrast was obtained for the order anal to vaginal to brief/partial intercourse (see Table 4).

Within each act type, simple effects group comparisons demonstrated three significant findings: In line with the moderation hypothesis, the gay male group rated anal intercourse significantly higher than both of the heterosexual groups (males *F*(1, 95) = 13.21, *p* < .001, *η*_p_^2^ = .12; females *F*(1, 173) = 11.71, *p* < .001, *η*_p_^2^ = .06); additionally, the heterosexual females rated brief/partial intercourse significantly higher than the heterosexual males, *F*(1, 186) = 12.14, *p* < .001, *η*_p_^2^ = .06.

## Discussion

### Graded Structure in Sexual Definitions

The primary prediction of the current research was that sexual definitions would display graded structure. As hypothesized, certain types of acts were judged to be significantly more constitutive of having “had sex” and of virginity loss than other types of acts. Definitions of sexual behavior should thus be considered as categorizations exhibiting graded structure, in common with a wide array of previously identified category types (e.g., Barsalou, 1985; Oakes, Haslam, & Turner, 1998; Rosch, 1974, 1978).

The current findings confirm the Horowitz and Spicer (2013) ratings hierarchy for definitions of sex and demonstrate a similar ratings hierarchy for definitions of virginity loss. They also support qualitative evidence concerning equivocal experience that falls outside of any simple inclusion–exclusion dichotomy in definitions of sex (Peterson & Muehlenard, 2007) and virginity loss (Carpenter, 2001). Such findings challenge the pervasive, implicit assumption in previous quantitative work that sexual definitions are a matter of dichotomous judgment (e.g., Sanders & Reinisch, 1999; Sanders et al., 2010; Sawyer, Howard, Brewster-Jordan, Gavin, & Sherman, 2007). Moreover, the graded structure approach offers a theoretical account for the robust between-subjects endorsement hierarchy for sexual definitions in previous research. In this account, broad agreement about degree of membership judgments results in a robust hierarchy of acts. Meanwhile, interindividual variation regarding category boundary placement (cf. Hampton, 2007; Verheyen & Storms, 2013) produces a correlation between the hierarchical position of an act and its likelihood of endorsement as constituting sexual behavior.

The graded structure hypothesis included two additional predictions regarding the order of behaviors in the definitional hierarchy. The first prediction was supported. Ratings of both sex and virginity loss evinced the anticipated hierarchy: intercourse acts; unidirectional (and nonpenetrative) genital contact acts; nongenital contact acts. These findings corroborate the endorsement rate hierarchy of Byers et al. (2009) for definitions of sex and abstinence (the latter in reverse order), as well as other studies in which intercourse acts receive significantly higher endorsement than oral-genital contact (Cecil et al., 2002; Trotter & Alderson, 2007). From a graded structure perspective, these findings suggest that a key criterion for goodness of membership judgments within categorizations of sexual behavior may be genital involvement. Further nuance of such a criterion is also observable from the current analysis. For definitions of both sex and virginity loss, PCA positioned nonpenetrative genital-to-genital contact with the unidirectional genital contact acts and placed the three genital self-stimulation acts with the nongenital contact acts. Such findings may be an indication that penetration and interpersonal physical contact play some part in such a genital involvement criterion. However, more direct testing would be required before the goodness of membership criteria pertinent to judgments of sexual behavior can be conclusively identified.

The second specific graded structure prediction was that significant differences would be found, positioning vaginal intercourse as the highest intercourse act, followed by anal intercourse, followed by brief/partial intercourse. This prediction was partially fulfilled. For definitions of sex, all four groups rated vaginal intercourse significantly higher than brief/partial intercourse but only three groups rated anal intercourse significantly higher than brief/partial intercourse. This difference was nonsignificant for the lesbian group. Meanwhile, for definitions of virginity loss, all the predicted significant differences emerged but only for three of the four groups—the exception being the gay male group. Before conclusions can be reached concerning this pattern of results, the study’s findings need to be considered in the light of the secondary, moderation hypothesis.

### The Moderating Role of Sexual Orientation Identity

As much as graded structure predicts global patterns of agreement within judgments of sexual behavior, it also predicts systematic variation in such judgments. The current research focused on moderation of generic graded structure in sexual definitions corresponding to sexual orientation identity—the combination of gender and sexual orientation among our four groups of participants.

The first of two predictions of the moderation hypothesis was thoroughly supported by the current findings: Ratings of unidirectional or nonpenetrative genital contact by the lesbian group were significantly upgraded with respect to both sex and virginity loss, compared to the other three groups. Such findings confirm and extend Horowitz and Spicer’s (2013) demonstration of significant differences in definitions of sex between their lesbian and heterosexual samples, for judgments of unidirectional genital contact acts.

The second prediction of the moderation hypothesis was that the gay male sample would upgrade their goodness of membership assessments of anal intercourse. This prediction was, on balance, partially supported. In terms of group differences, only for definitions of virginity loss did the gay male group rate anal intercourse significantly higher than other groups and, even then, this was only compared to the two heterosexual groups—not the lesbian group. No such group differences emerged for definitions of sex. However, other indications did support the prediction. Within definitions of both sex and virginity loss, the gay male group rated anal intercourse highest of all the behaviors and significant linear contrasts emerged for the gay male group in the order anal to vaginal to brief/partial intercourse. This top rating position for anal intercourse coheres with the higher endorsement rates of anal over vaginal intercourse among gay males found by Hill et al. (2010). Additionally, for virginity loss definitions, though not for definitions of sex, ratings by the gay male group deviated from the pattern of the other three groups, among whom vaginal intercourse was rated significantly higher than anal intercourse and anal intercourse was rated significantly higher than brief/partial intercourse.

Taken together, these findings largely support the prediction of the current research that the generic graded structure of sexual definitions would be subject to group-level moderation corresponding to sexual orientation identity. Specifically, we predicted and found evidence of upgraded ratings of the most elevated identity-congruent sexual behavior, among those for whom vaginal intercourse (the generically most highly rated exemplar) is sexual orientation identity-incongruent. This pattern of upgrading was clear-cut with respect to the lesbian sample but only partial with respect to the gay male sample.

Also relevant was that gay male ratings for sex of unidirectional or nonpenetrative contact were significantly upgraded compared to the heterosexual males. This suggests that group-level moderation of graded structure may not be exclusively limited to the most highly rated behavior that is sexual orientation identity-congruent.

In the current research, the prediction that sexual orientation identity would play a moderating role in sexual definition judgments was founded upon a set of assumptions about how such identities are experienced and enacted. Research into sexual behavior has highlighted differences in the sexual experiences of lesbians, gay men, and heterosexuals of both genders (e.g., Blair & Pukall, 2014; Kinsey et al., 1953; Lever, 1995; Savin-William, 1990). Meanwhile, categorization researchers have shown contextual perspective and episodic knowledge to affect typicality judgment and exemplar generation (e.g., Barsalau & Sewell, 1984; Vallée-Tourangeau et al., 1998). The pattern of between-groups differences found here is, therefore, interpreted as proceeding, at least in part, from differential sexual experience, contextual perspective, and episodic knowledge between the sexual orientation identity groups. It should be noted, however, that the current research undertook no direct investigation of the potential mechanisms of variation driving the group differences demonstrated here. Further research is clearly needed.

We additionally propose that the group differences demonstrated in the current research may reflect identity-management practices (cf. Horowitz & Spicer, 2013): in particular, shared methods by which sexual orientation identities are embraced or resisted (cf. Carpenter, 2001). We would argue that, in addition to any automatic mechanisms by which goodness of membership criteria, sexual experience, contextual perspective, and episodic knowledge are activated, each would also be amenable to rhetorical deployment in the interests of identity-management. In this way, we contend that a graded structure perspective can provide a framework for further exploration of the motivational, rhetorical, and consequence-sensitive dimensions (cf. Peterson & Muehlenard, 2007) of sexual definitions.

### Study Limitations

The present findings must be considered in the light of limitations of the study. Firstly, the current sample was dominated by heterosexual females, who made up 49% of the participants, while 21% of participants were heterosexual males, 15% were lesbians, and 16% were gay males. Conclusions from the current sample concerning the main effect of gender and/or sexual orientation should therefore be considered with some caution. However, the three-way interactions, which form the central findings of the present research, demonstrated graded structure within each of the sexual orientation identity groups sampled, ensuring that graded structure was not an artifact of uneven group sizes. Neither was the imbalance between the groups relevant to testing of the moderation hypothesis. Nevertheless, replication with a more balanced sample may be advisable, in order to fully unpack the relative importance for sexual definitions of gender, sexual orientation, and the interaction of the two. Additional features of our sample should also be attended to. Recruitment was targeted at the age range of 18–30 years, and participants were predominantly students, and almost exclusively British. Caution is thus recommended before generalizing the findings to other age groups and nationalities or to those of lower educational attainment.

Secondly, in the current research, participants were asked to rate the list of acts with respect to their own behavior. This may have elicited a different pattern of response than if we had asked for ratings of a third party’s behavior. For example, it might have focused participants on their experience and/or episodic knowledge, especially for acts that the participant has, versus has not, practiced. If so, this may have influenced the group differences findings. For example, lesbians’ ratings of unidirectional and nonpenetrative genital contact and gay males’ ratings of anal intercourse might have been informed more by actual experience than those of the other groups. Identity-management effects are also likely to be sensitive to whether one’s own or a third party’s behavior is being rated. In sum, we can be less confident of finding the current pattern of group differences for third-party judgments of sexual behavior without a thorough testing of such cue effects.

Thirdly, a ceiling effect may be in operation with respect to the measurement of definitions of vaginal and anal intercourse. This may be a consequence of the six-point ratings scale used in the current research. Future research employing a sliding scale response methodology may capture additional nuance in the conceptualization of intercourse acts, shedding a clearer light on whether or not these acts are differentially defined.

### Conclusion

The current research has demonstrated graded structure in definitions of sex and virginity loss. A preliminary investigation of variability in goodness of membership judgments also showed strong indications of systematic definitional variation, in line with sexual orientation identity. These findings permit research into sexual definitions to move from a largely descriptive enterprise, to one capable of predicting and explaining the variability of sexual definitions. Such a development offers great potential toward improving the attunement of researchers and practitioners to the sexual definitions of the people they intend to study and/or help. It may also aid in identifying where variable definitions can arise that are associated with a range of societal ills. For example, by articulating the experiential, motivational, and contextual drivers of variable categorization, research employing a graded structure approach may recommend preventative and remedial measures with respect to potential sites of conflict (e.g., relationship breakdown, stigma) and risk (e.g., sexual health risk, sexual assault risk).

Pursuant to such ends, it is vital for future investigation to examine other moderators of graded structure than those focused upon here. At a group level, these might include, for example, relationship status, ethnicity, or HIV status (see, for example, Rawlings, Graff, Calderon, Casey-Bailey, & Pasley, 2006). Moreover, the proposed drivers of definitional variation should also produce both cultural (e.g., collectivist vs. individualist cultures, religious vs. secular societies) and interindividual variation (e.g., differences in personality, sexual experience, sex education, sociosexuality), which could usefully be explored in the future. In particular, motivated variation in sexual definitions is likely to be relevant to a range of applied fields, such as health (e.g., definitions of safe sex), counselling (e.g., definitions of infidelity), and forensic contexts (e.g., definitions of rape), and therefore merits careful study.

## Appendix

See Table 5.

**Table 5:** Mean ratings and SDs for definitions of having “had sex” and virginity loss

	Sex (*n* = 260)		Virginity loss (*n* = 257)	
Sexual behavior	*M*	*SD*	*M*	*SD*
*Intercourse*				
Vaginal intercourse	5.94	.42	5.84	.82
Anal intercourse	5.68	.91	5.06	1.57
Brief/partial intercourse	5.16	1.12	4.97	1.46
*Unidirectional/nonpenetrative genital contact*				
Receptive sex aid contact	3.87	1.70	2.92	1.92
Initiative oral-genital contact	3.87	1.74	2.34	1.78
Initiative sex aid contact	3.84	1.73	2.67	1.91
Receptive oral-genital contact	3.81	1.77	2.52	1.86
Nonpenetrative genital-to-genital contact	3.18	1.65	2.24	1.64
Initiative manual-genital contact	2.96	1.57	1.85	1.38
Receptive manual-genital contact	2.90	1.56	1.91	1.43
*Nongenital contact*				
Simultaneous masturbation in another’s presence	2.39	1.45	1.63	1.15
Simultaneous masturbation via phone contact	1.87	1.21	1.33	.88
Simultaneous masturbation via computer contact	1.81	1.19	1.34	.85
Initiative oral contact with breasts/nipples	1.78	1.19	1.35	.86
Initiative manual contact with breasts/nipples	1.73	1.13	1.32	.80
Receptive manual contact with breasts/nipples	1.69	1.07	1.33	.80
Receptive oral contact with breasts/nipples	1.69	1.10	1.25	.72
Deep kissing	1.21	.67	1.12	.58

Absolute range for ratings of both sex and virginity loss was 1–6
